# A remark on copy number variation detection methods

**DOI:** 10.1371/journal.pone.0196226

**Published:** 2018-04-27

**Authors:** Shuo Li, Xialiang Dou, Ruiqi Gao, Xinzhou Ge, Minping Qian, Lin Wan

**Affiliations:** 1 School of Mathematical Sciences, Peking University, Beijing, China; 2 National Center of Mathematics and Interdisciplinary Sciences, Academy of Mathematics and Systems Science, Chinese Academy of Sciences, Beijing, China; University of Helsinki, FINLAND

## Abstract

Copy number variations (CNVs) are gain and loss of DNA sequence of a genome. High throughput platforms such as microarrays and next generation sequencing technologies (NGS) have been applied for genome wide copy number losses. Although progress has been made in both approaches, the accuracy and consistency of CNV calling from the two platforms remain in dispute. In this study, we perform a deep analysis on copy number losses on 254 human DNA samples, which have both SNP microarray data and NGS data publicly available from Hapmap Project and 1000 Genomes Project respectively. We show that the copy number losses reported from Hapmap Project and 1000 Genome Project only have < 30% overlap, while these reports are required to have cross-platform (e.g. PCR, microarray and high-throughput sequencing) experimental supporting by their corresponding projects, even though state-of-art calling methods were employed. On the other hand, copy number losses are found directly from HapMap microarray data by an accurate algorithm, i.e. CNVhac, almost all of which have lower read mapping depth in NGS data; furthermore, 88% of which can be supported by the sequences with breakpoint in NGS data. Our results suggest the ability of microarray calling CNVs and the possible introduction of false negatives from the unessential requirement of the additional cross-platform supporting. The inconsistency of CNV reports from Hapmap Project and 1000 Genomes Project might result from the inadequate information containing in microarray data, the inconsistent detection criteria, or the filtration effect of cross-platform supporting. The statistical test on CNVs called from CNVhac show that the microarray data can offer reliable CNV reports, and majority of CNV candidates can be confirmed by raw sequences. Therefore, the CNV candidates given by a good caller could be highly reliable without cross-platform supporting, so additional experimental information should be applied in need instead of necessarily.

## Introduction

Copy number variations (CNVs) are gain and loss of DNA sequence of a genome, which can result in DNA structural variations of the individual or the cell. CNVs play important roles in the genomic mechanism of diseases, including non-small cell lung cancer [1], systemic lupus erythematosus and similar inflammatory autoimmune disorders [2], autism [3–6], schizophrenia [3, 7] and idiopathic learning disability [8]. In cancer diagnosis and prognosis, CNVs are even more important and frequently studied. They can be used as biomarkers to predict cancer status and locate cancer origin.

The application of CNVs as biomarkers requires accurate experimental and computational methods for calling CNVs. With rapid development of experimental technologies, more and more studies pay attention to disease related CNVs. Genome wide CNV identification relies on various approaches, among which SNP microarray and DNA sequencing are classic high throughput platforms.

Apart from the development of experimental technologies, computational approaches for calling CNVs are also developed and improved. Despite of the improvement of computational approaches, it is generally believed that after calling from high throughput methods, the CNV candidates are not reliable enough to use. A cross-platform validation, such as the polymerase chain reaction (PCR) is usually carried out after CNV candidates are called computationally. Majority of CNV reports published or utilized in scientific studies is filtered using extra independent experimental information. However, no studies show the low reliability of CNV candidates called by computational tools from microarray and NGS, while no studies show the effect of cross-platform validation on computationally called CNV candidates.

In this study, we are aimed to assess the reliability of computational tools for CNV calling without the effect of cross-platform validation. Raw sequencing data are regarded as the golden standard of a genome. To figure out the difference of CNV reports with and without experimental validations, the microarray data from Hapmap Project were utilized. An accurate CNV calling algorithm of Affymetrix SNP array, CNVhac [9], was applied to detect CNVs. First, we compared the copy number loss reports released by Hapmap Project using microarray data [10, 11] and 1000 Genomes Project using NGS data [12, 13], both of which have cross-platform supporting from different experimental methods. We showed that the overlap of these two reports is small (less than 30%). The inconsistency between different reports might result from different information contained in microarray data and NGS data, different criteria for CNV calling, or from the filtration effect of extra independent experimental information. NGS data are considered to have more information than microarray data, in the aspect of genome coverage. Therefore, we checked the reliability of CNV candidates called from microarray data. We found that for almost all of copy number loss candidates called from microarray by CNVhac have lower read mapping depth on the independent NGS data, indicating real copy number losses. Furthermore, 88% copy number loss candidates have corresponding breakpoints found in raw sequences in NGS data. Such copy number loss candidates with corresponding breakpoints appearing in raw sequences are strong evidence showing the high reliability of CNVs called by microarray data with a good caller. But almost a half of these copy number loss candidates are in neither list of the two reports. Thus, a possible reason of exclusion of those copy number losses is the introduction of false negative results from the requirement of the additional cross-platform supporting. This indicates that the calling from microarray data could be highly reliable when a good caller is applied, and we should be careful with the results with extra experimental information input at that time.

## Materials and methods

### Data

The Affymetrix SNP 6.0 array data from Hapmap Project was used in this study. CNVhac algorithm was applied to detect CNVs [9]. Besides, Hapmap Project also released the CNV reports which have cross-platform supporting from different experimental methods. A total of 254 human DNA samples (see S1 File) in the Hapmap Project were also DNA sequenced by NGS independently from the 1000 Genomes project. Three samples (NA06991, NA06993 and NA07029) have a ultra-large number of CNVs comparing to others (more than one magnitudes larger), so they were removed in the following analyses. The sequence read mappings to Chromosome 11 and Chromosome 20 were released the 1000 Genomes projected. Thus, we obtain the mapped DNA sequencing data of corresponding samples from 1000 Genomes project. Besides the read mapping profile, the 1000 Genomes project also provided various genetic variants called based on the DNA sequencing data, which were released in variants calling format (VCF) files.

In summary, we have obtained a CNV calling result without experimental confirmation, which is called by CNVhac based on SNP microarray data released by Hapmap Project, and two CNV reports, one released from Hapmap and one report from the1000 Genomes Project, both of which have experimental validation (Supplementary material in [9] and [12]). We used the Affymetrix SNP array data of 254 samples (ftp://ftp.ncbi.nlm.nih.gov/hapmap/raw_data/hapmap3_affy6.0/) and released CNV reports (ftp://ftp.ncbi.nlm.nih.gov/hapmap/cnv_data/) from Hapmap Project, and the VCF files and chromosome 11 alignment BAM files of 128 samples (ftp://ftp-trace.ncbi.nih.gov/1000genomes/ftp/release/20130502/ and http://ftp.1000genomes.ebi.ac.uk/vol1/ftp/phase3/data/) from 1000 Genomes Project. We preprocessed the raw data instead of putting them into use directly.

### Data preprocessing

The data on Hapmap is using UCSC hg18 annotation while data on 1000 Genome is using UCSC hg19 annotation. To compare results from the two projects, we transformed all results from Hapmap data to UCSC hg19 on UCSC Genome Browser. To compare different results from microarray and sequencing, we selected the copy number losses in DNA sequencing results where the regions contain ≥ 5 SNP probe sets in Affymetrix SNP 6.0 array, since CNVhac requires at least 5 probe sets to make a CNV call. Also to avoid highly repetitive and complex regions, only CNVs overlapped with gene regions were involved. The gene regions are defined in UCSC hg19 annotation as well. The Samtools [14] was used to extract the read mapping depth information from 1000 Genomes for our testing. For copy number losses in 1000 Genome project, we extracted variants with ALT value in {CN0, DEL}.

### Statistical testings on read depth

The mapping results of chromosome 11 was used to confirm that the reported copy number loss region from CNVhac [9] from microarray has a relatively low read mapping depth [14]. We used two hypotheses testing to show that the assertion is true; the *χ*^2^ test and permutation test. The *χ*^2^ test is a widely-used test to check if two discrete distribution are same or not, and permutation test conducts the two-sample test when the distribution is unknown. Here the permutation test was used to further confirm read depth in copy number loss region is lower than other parts of genome. Let *F* denote the read depth distribution of reported region and let *G* denote the normal read distribution, and the null-hypothesis is:

$$\begin{array}{c} H_{0}:F=G. \end{array}$$

As to this specific problem, because we only tested the region reported as copy number loss, and thus our goal was to verify that the reported region has a relatively low read depth. We changed the original test to: let *μ* denote the average read depth in the reported region and let *σ* denote the mean read depth form normal region. Then the null-hypothesis is:

$$\begin{array}{c} H_{0}:\mu \geq \sigma . \end{array}$$

We first sampled an equal length of read depth from the whole chromosome. Due to the long length of the region, it is safe to assume that the sampled set has the normal read depth distribution. Then we resampled the combination of the set of reported regions and the just sampled set with the same length for 999 times and computed the average read depth *σ*_*i*_, *i* = 1, 2, …, 999. Let *σ*_(1)_, *σ*_(2)_, …, *σ*_(999)_ denote the ascend order statistics. Suppose there exists *i* such that *σ*_(*i*+1)_ < *μ* ≤ *σ*_(*i*)_ then *p* = *i*/1000, otherwise *p* = 1. A threshold will be taken to select the copy number loss candidate.

**Fig 1:**
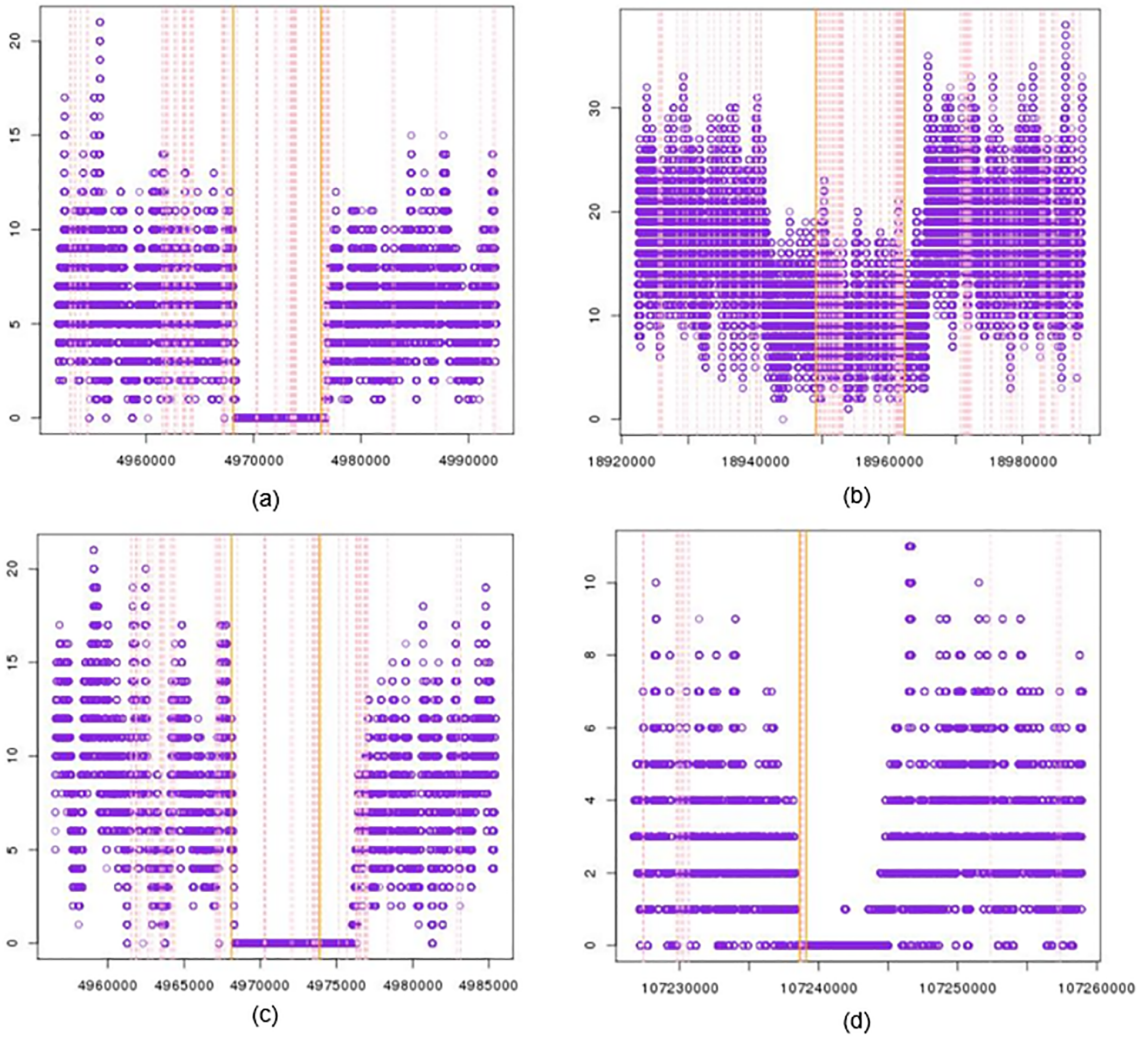
Plots of copy number loss candidates and their adjacency. In each plot, x-axis shows the loci on the Chromosome 11; y-axis shows the read depth; purple dot is read depth for corresponding loci; orange vertical line is the reported copy number loss boundary; pink dashed line is the loci of probe on SNP microarray.

To gain an intuitive sense of the regions, plots of read depth of copy number loss regions along with their adjacent regions are expected to be in a concave shape (Fig 1), which can prove the drop of read depth in the reported regions. Thus, the same two testings on the copy number loss regions with their left and right adjacent were also performed.

### Breakpoint seeking method

To find the breakpoint of sequence in the reference genome, we used two techniques to recover the adjacent sequence of the copy number loss candidates, purely assembling and Cufflinks [15]. Reads covering the adjacent regions of the copy number loss and unmapped reads are first extracted, and de novo assembled. Like the idea of split-read methods from sequence structure variation detection, unmapped reads are supposed to possibly containing a breakpoint. De Bruijn graphs are used to assemble reads. Contigs obtained from de novo assembly are further compared to the reference genome. Contigs which are neighbors on the de Bruijn graph, but are far away from each other on the reference genome, are considered covering aberrant genome structures, i.e. copy number losses here. Purely assembling can provide us the most convincing evidence with no doubt, because reads with breakpoints are caught. Due to the low depth (about ×4) of the released mappings, only considering purely assembling might make lots of true copy number losses left out. We utilized it only on those candidates that fail to be found by purely assembling. For each contig, we used Cufflinks, to be specific, the sequence mapping component TopHat, to find breakpoints. TopHat is a fast mapper allowing splice junction. A splice junction in a gene region is similar to a copy number loss junction in our CNV region, since the intron would be cut off in splicing, where reads is just like resulted from a missing genome region in a copy number loss. Contigs were mapped to the related expanded CNV region using Cufflinks, and copy number loss breakpoints would be returned in the same way where the splice junctions would be identified between exons in RNA-Seq analysis.

## Results

### Low consistency of different CNV calling results

Hapmap Project provided a CNV calling report based on SNP microarray data using QuantiSNP [16] and Birdseye [17]. The 1000 Genome Project also provided a DNA structural variation report as a VCF file based on NSG data. Both reports were published with extra experimental information input. We found that the samples involved in 1000 Genome Project have an overlap of 254 samples with Hapmap Project, which offered an opportunity to compare the results from microarray and NGS.

Since the copy number loss can be checked by finding the break sequences from NGS, here we only compared the copy number losses in the two reports on the whole genome scale.

Due to the restriction of location of probe sets on microarray chips, using microarray data we usually cannot see relatively small copy number losses (compared to the distance between probe sets) and may miss the boundaries of copy number losses. To have a fair comparison, we exclude copy number losses from NGS containing less than 5 probe sets in VCF file. Because of the region found for a same CNV can be different by different methods, we regard two CNVs detected by different approaches as the same if the two CNVs have at least 50% base pair overlap. Surprisingly, we found that the two reports shared a small proportion (less than 30%) (see Table 1).

**Table 1:** Comparison of copy number loss from Hapmap Project and 1000 Genome Project.

Chrom	Hapmap	Hap. over.	%	Seq	Seq. over.	%
11	998	89	8.92%	756	192	25.39%
whole genome	16493	2846	17.26%	9305	2517	27.05%

We denote the number of copy number losses reported by 1000 Genome Project as “Seq”, the part of which overlapping with Hapmap report by Seq.over and the percentage of the overlapping proportion as “%”; while copy number losses from Hapmap Project as “Hapmap”, the part of which overlapping with 1000 Genome report as “Hap. over.” and percentage of overlapping part as “%”

We then calculated the percentage of overlapped copy number losses between report of CNVhac and the report of 1000 Genome Project, as well as the percentage between report of CNVhac and report of Hapmap Project. Table 2 is the comparison with the copy number losses called by CNVhac from microarray and copy number losses from NGS in VCF file. Less than 20% of the regions reported by CNVhac have overlaps with the copy number losses called by NGS data. Further, we found that if two copy number loss regions from CNVhac and VCF file overlap, the overlap lengths are quite long (average: 26170bp, mode: 10369bp), and the ratio of overlap length to CNV length is closed to 1 in most cases. Table 3 is the comparison with the copy number losses called by CNVhac from microarray and copy number losses released by Hapmap Project. About 50% regions detected by CNVhac have overlap with copy number losses in Hapmap Project reports; less than 20% of copy number losses in Hapmap Project reports share base pairs with copy number losses called by CNVhac.

**Table 2:** Comparison of copy number losses from 1000 Genome Project and CNVhac calling.

Chrom.	CNVh.	CNVh. over.	%	Seq	Seq. over.	%
11	281	46	16.37%	756	70	9.26%
whole genome	5185	750	14.46%	9305	955	9.19%

We compare them in two scales: chromosome 11 (see S2 File) and the whole genome. We denote the copy number loss candidates reported by CNVhac based on SNP microarray as “CNVh.CNV”, and denote that called by sequencing as “Seq.CNV”. In each scale, “CNVh.” column is the total number of “CNVh.CNV”, “CNVh. over.” is the number of “CNVh.CNV” shared no less than 50% base pairs with some “Seq.CNV”; “Seq.” is the total number of “Seq.CNV”, “Seq. over.” is the number of “Seq.CNV” shared no less than 50% base pairs with some “CNVh.CNV”. “%”column is the ratio of overlap cases to the total number in “CNVh.” and “Seq.” respectively. Note that the difference between “Seq. over.” and “CNVh. over.” causes by multiple copy number loss candidates from one method matching to a single candidate from the other method.

**Table 3:** Comparison of copy number losses from Hapmap Project and CNVhac calling.

Chrom.	Hapmap.	Hap. over.	%	CNVh.	CNVh. over.	%
11	998	113	11.32%	281	145	51.60%
whole genome	16493	2726	16.53%	5185	2758	53.19%

We also compare them in two scales: chromosome 11 (see S2 File) and the whole genome. We denote the copy number loss candidates reported by CNVhac based on SNP microarray as “CNVh.CNV”, and denote CNVs from Hapmap Project as “Hapmap.CNV”. In each scale, “CNVh.” column is the total number of “CNVh. CNV”,“CNVh. over.” is the number of “CNVh. CNV” shared no less than 50% base pairs with some “Hapmap. CNV”; “Hapmap.” is the total number of “Hapmap. CNV”, “Hap. over.” is the number of “Hap. CNV” shared no less than 50% base pairs with some “CNVh. CNV”. “%” column is the ratio of overlap cases to the total number in “CNVh.” and “Hapmap.” respectively. Note that the difference between “Hap. over.” and “CNVh. over.” causes by multiple copy number loss candidates from one method matching to a single candidate from the other method.

With such results, it is natural to ask, whether this happens due to the information implicated in these two types of data is very different, or the effect of different detection criteria used including multiple cross-platform supporting? In the following analysis we will show that copy number loss calling from microarray can be validated by low read depth as well as the break sequences in corresponding NGS data.

### Statistical tests on the low read mapping depths of copy number loss regions called by CNVhac

People generally believe microarray data contain less information than NGS data in the aspect of genome coverage. We then tested the copy number loss in microarray data in Hapmap called by CNVhac [9] using the read mapping depths information of the independent NGS data from the 1000 Genome Project. If the copy number loss can be confirmed in raw sequences, we can believe that microarray data are reliable for CNV calling. We verified the CNV candidates by 2 kinds of statistical testing, *χ*^2^ test and permutation test. From the 1000 Genome project, we obtained read mapping profile of 128 samples in Chrom 11 and the corresponding sequencing read depth.

Table 4 shows our results of hypothesis testing. All but two samples accept null hypothesis, which indicates that most reported region have a relatively low read depth compared to normal regions. Besides, the two remained cases passed the *χ*^2^ test but showed a *p*-value of 1 in the permutation test. A *p*-value of 1 also indicates an abnormal case in permutation test: this region has a relatively high read depth. This phenomenon might be caused by other structure variation or mutation other than copy number loss.

**Table 4:** Statistical testings of the reported region with the whole chromosome, only two samples accept the null hypothesis.

test	*p* < 0.01	*p* < 0.05
*χ*^2^ test	100%	100%
permutation test	99.10%	99.10%

We plotted the read depth in the region of copy number loss candidates and their adjacency. Fig 1 shows the read depth near the reported regions. The orange vertical line interval shows the reported area and the pink lines give the position of probes. Those concave figures confirm that the reported area indeed have a drop of read depth. In 128 samples, most plots are similar with (a) or (b), which directly proves a drop of read depth in the reported region, thus it gives a reasonable copy number loss region. In (a) the read depth in middle is almost exact zero, and in (b) the middle part has an obvious low read depth. Yet there are some like (c) or (d), there is still a low depth region exceeding the reported region, which reflects our initial observation of microarray drawback. We should also suggest that the zero depth is not caused by centromere or other reasons, which make it undetectable, because we examined the case NA12004 that shows a strictly positive read depth on those regions.

We also tested on the reported region with its adjacent area, aiming to test a concave shape of read depth in the region and to show a drop of read depth in the reported region. The *χ*^2^ tests and permutation tests performing on copy number loss candidates with their adjacent regions (Table 5). All suspect copy number losses rejecting the null-hypothesis in both tests also reject the null-hypothesis in tests for the whole genome. Tests held on both whole genome and adjacent regions depict that the reported copy number loss region has a relatively low read depth. Nearly all the *p*-values are significant in testing with adjacent region. The testing results are slightly weaker compared to the one tested in the whole chromosome, but it still gives convincing results. The lower read depth of the reported region compared to the normal region supported our observation that even a copy number loss reported by CNVhac is not a complete copy number loss; it might be part of a copy number loss or has some mutations. Thus, in scientific research, it is reasonable to further study these abnormal regions in other aspects.

**Table 5:** Statistical testings of the reported region with its adjacent area.

test	*p* < 0.01	*p* < 0.05	NA
*χ*^2^ test for upstream	99.10%	99.10%	0%
*χ*^2^ test for downstream	93.60%	93.60%	0%
permutation test for upstream	96.60%	96.60%	0.90%
permutation test for downstream	89.80%	89.80%	6.40%

The first and third rows are tests for upstream; others are tests for downstream. Here NA is caused by zero depth in whole reported region.

In conclusion, almost all suspect copy number losses are reasonable according to a obvious relatively low read depth in corresponding region.

### High detection rate of breakpoints in copy number loss candidates detected from microarray

**Fig 2:**
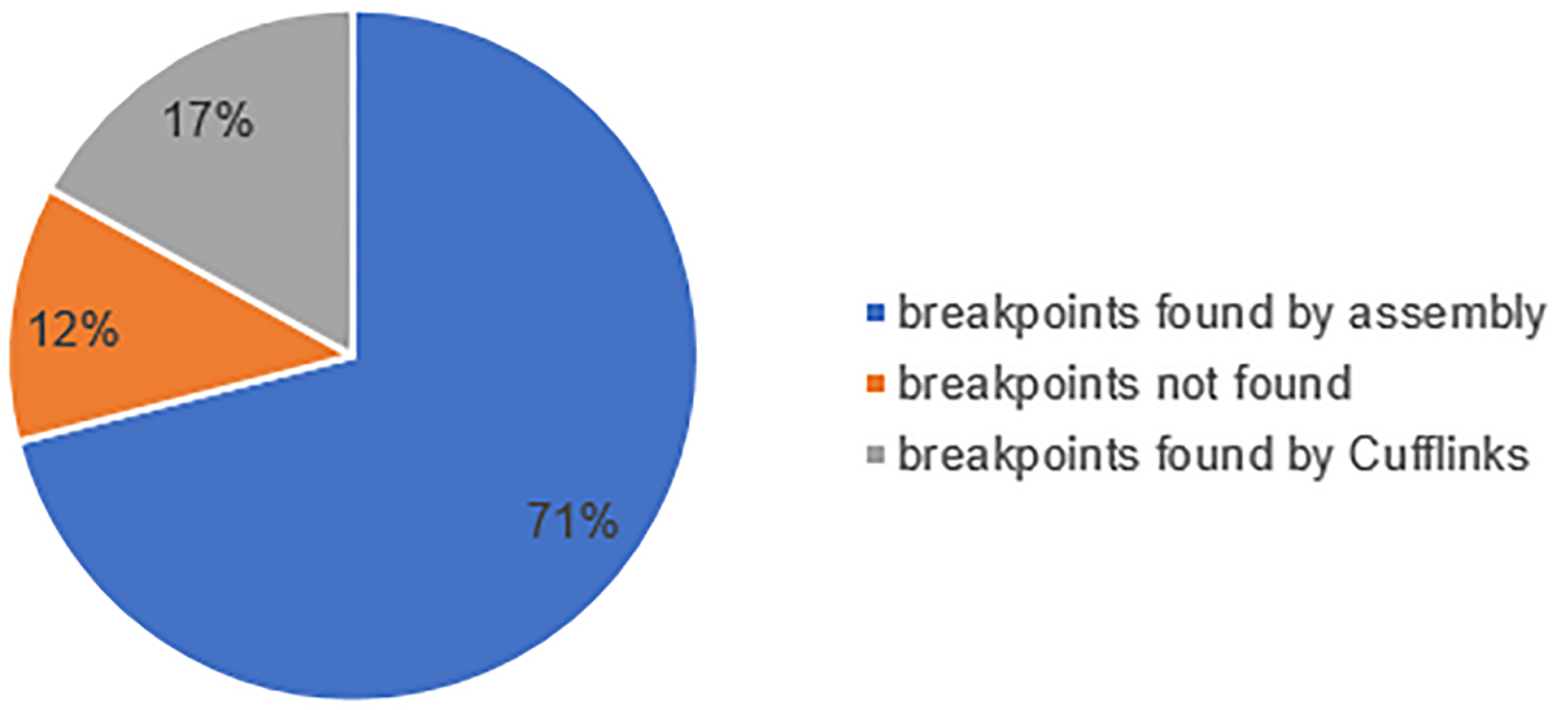
Pie plot of results in seeking breakpoints on deletion candidates and its adjacent area by the breakpoint seeking method.

We then searched the breakpoints of sequence in copy number loss which are the joints of two ends of these candidate copy number losses for a stronger support of the CNVhac callings. To verify the reliability of copy number losses, the read mapping files released on the FTP of 1000 Genome project were involved in further analysis. The data are limited in the aspects of region on the genome and number of samples. The data for chromosome 11 are the most abundant, so we used the 128 alignment files of chromosome 11 accessible on 1000 Genome Project. All 128 samples are contained in the 254 samples involved in the early analysis. In the copy number loss report of CNVhac, there are 235 copy number losses in total from chromosome 11 of the 128 samples. We used human reference sequence GRCh37 produced by the Genome Reference Consortium as our reference sequence. Due to missing of some certain regions of the reference, we examined 207 of total 235 copy number losses. Using the breakpoint seeking method in **Methods** section, we found that 88% of candidate copy number losses have solid evidences from NGS data, and among them 71% of them has even direct evidences of break reads (Fig 2).

Up to now, we found solid evidence for copy number loss candidates detected from microarray data by CNVhac. Hence, although microarray data contain less information, it is still reliable for CNV calling with high accuracy. The inconsistency of CNV reports in two public databases is likely resulted from the inconsistent detection criteria as well as the different multiple cross-platform validation.

## Discussion

With the development of high throughput biotechnologies and CNV calling algorithms, CNV identifications are playing an important role in disease mechanism and personal medicine. Despite of the progress, a common sense seems to exist in academic community that CNVs directly detected by present CNV calling algorithms have a high false discovery rate and are unreliable. In this study, we compared two current authoritative CNV reports, and utilized read depth as well as the breakpoint seeking method, to verify the copy number loss candidates from a good CNV caller, e.g. CNVhac, are highly reliable.

We first compared copy number loss report released by Hapmap Project and 1000 Genome Project. With reasonable preprocessing, copy number loss reports, which are expected to be similar, contradict each other. The controversial fact raises questions towards our current filtration of CNV candidates. Then we further verified a set of copy number loss called directly from microarray data provided in Hapmap Project. Over 71% of candidates are confirmed by sequences containing breakpoints. These candidates are no doubt to be the true copy number losses. Almost 17% of candidates have quite high potential to be the true candidates, because of paired end reads detected using cufflinks. With 88% true copy number losses, we can conclude the copy number loss candidates are relatively trustable even without experimental validation. We also compared the copy number losses called by CNVhac with the two authoritative CNV reports. Less than 55% copy number losses we found are occurred in either CNV report of Hapmap Project or variant calling file of 1000 Genome Project. Here we can infer that the inconsistent detection criteria as well as the different multiple cross-platform validation lead to contradict CNV reports of the same person.

## Conclusion

Up to now, we raise questions towards the quality control methods of CNV calling results. The inconsistent CNV reports from both Hapmap Project and the 1000 Genome Project arouse our doubt towards the current quality control method of CNV calling results, including post-filtration and cross-platform supporting. We analyzed the copy number loss candidates via statistical analysis on read depth from NGS as well as a breakpoint seeking method using sequence alignment data, at least 88% of candidates are highly reliable, which question the experimental validation. We suggest current experimental validation may have a high false exclusion rate when validating CNV candidates. The quality control method should be adjusted according to the CNV calling method used. The CNV candidates given by a good caller could be highly reliable without cross-platform supporting, so additional experimental information should be applied in need instead of necessarily.

## Supporting information

S1 File Sample ID included in the study. The file includes sample IDs of 254 subjects, so that all raw data can be retrieved on the official webpage of the two projects. The 251 subjects involved in analyses are highlighted.
(XLSX)

S2 File Copy number losses on chromosome 11 from the three sources. The file includes three sheets, which contains the copy number losses published by Hapmap Project, 1000 Genome Project, and detected by CNVhac.
(XLSX)
